# Conservative Management of Unstable Angina With Incidental Coronary‐Pulmonary Artery Fistula: A Case Report

**DOI:** 10.1002/ccr3.71044

**Published:** 2025-10-10

**Authors:** Jia‐jun Zhang, Xiao Li, Jin‐long Zhang, Hai‐tao Zhang, Fei‐fei Su

**Affiliations:** ^1^ Department of Cardiovascular Medicine Air Force Medical Center, Chinese People's Liberation Army Beijing Beijing China; ^2^ China Medical University Shenyang Liaoning Province China; ^3^ Graduate School of Hebei North University Zhangjiakou Hebei Province China; ^4^ Department of Radiology Air Force Medical Center, Chinese People's Liberation Army Beijing Beijing China

**Keywords:** case reports, coronary angiography, coronary computed tomography angiography, coronary heart disease, fistula of coronary‐pulmonary artery

## Abstract

Coronary‐pulmonary artery fistula (CPAF) is a rare cardiovascular condition that requires precise diagnosis through comprehensive multimodal imaging studies; surgical intervention is not always indicated for its management.

## Graphical abstract

Coronary angiography showed 50% LAD stenosis (yellow arrow), malformed proximal LAD vessels (red arrows), and blood shunting to the pulmonary artery (PA, blue arrow; white arrows for flow).

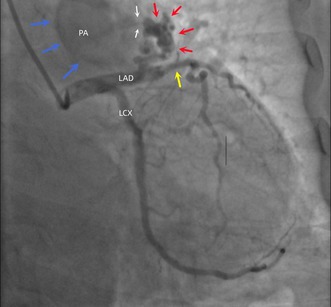

## 1 Introduction

We report a 65‐year‐old female admitted with unstable angina who incidentally had a coronary‐pulmonary artery fistula (CPAF) found during coronary angiography (CAG). After careful evaluation, we opted not to close the fistula. Postoperative comprehensive imaging confirmed the appropriateness of this conservative decision, and the patient's symptoms were effectively controlled with medical therapy. We hope this case will aid clinicians in recognizing and managing this rare condition, potentially improving treatment outcomes in similar scenarios.

## 2 Case Presentation/Examination

### 2.1 Chief Complaints

A 65‐year‐old female patient presented to our hospital with “recurrent episodes of dizziness, chest tightness, and palpitations.”

### 2.2 History of Present Illness and Past Illness

The patient reported recurrent symptoms for over 6 months, occurring in 3–4 episodes monthly. Each episode had no apparent cause, and the patient did not experience discomforts such as chest pain, headache, difficulty breathing, acid reflux, or dizziness during the episodes. The episodes lasted for approximately 3–5 min and resolved on their own.

The patient has a 20‐year history of hypertension and has been regularly taking nifedipine controlled‐release tablets and amlodipine for treatment, with blood pressure controlled around 140/90 mmHg; she also has a history of diabetes for more than 10 years and regularly takes repaglinide for treatment.

She denies having a history of heart disease or coronary artery disease, and there is no family history of such conditions.

### 2.3 Physical Examination

On admission, her blood pressure was 167/92 mmHg, and her heart rate was 76 beats per minute. Physical examination was unremarkable, with no cardiac murmurs or lung rales noted.

## 3 Methods

### 3.1 Differential Diagnosis

Her principal diagnosis is unstable angina, attributed to her comorbidities of hypertension and diabetes. Differential diagnoses for unstable angina include acute chest pain conditions such as acute myocardial infarction, aortic dissection, and pulmonary embolism.

Other possibilities to consider include arrhythmias (such as tachyarrhythmias, atrioventricular block, etc.), respiratory diseases (such as chronic obstructive pulmonary disease, chest tightness variant asthma, etc.), and neurological diseases (such as cerebral infarction, transient ischemic attack, etc.).

Additionally, other systemic diseases such as gastroesophageal reflux disease, hyperthyroidism, hypoglycemia, anemia, and chronic obstructive pulmonary disease also need to be considered, but they can essentially be ruled out after further inquiry into the patient's medical history and completion of examinations.

### 3.2 Investigations

Laboratory test results, including myoglobin (Mb), high sensitivity cardiac troponin T (Hs‐cTnT), creatine kinase MB isoenzyme (CK‐MB), D‐Dimer, and other routine biomarkers, were within normal limits. See Table 1.

**TABLE 1:** Laboratory examination data.

	Measured value (ng/mL)	Normal range (ng/mL)
Mb	27.40	25–58
Hs‐cTnT	0.01	0–0.014
CK‐MB	1.94	0–4.88
D‐Dimer	160.00	0–255

The electrocardiogram (ECG) revealed left ventricular hypertrophy, transthoracic echocardiography (TTE) demonstrated reduced left ventricular diastolic function with normal systolic function (ejection fraction: 68%).

Although the patient's laboratory results, ECG, and TTE were largely normal, her symptoms were clearly consistent with unstable angina. Given that long‐standing hypertension and diabetes probably caused coronary stenosis, we elected to perform CAG to define her coronary anatomy and allow prompt relief of any obstruction.

**FIGURE 1:**
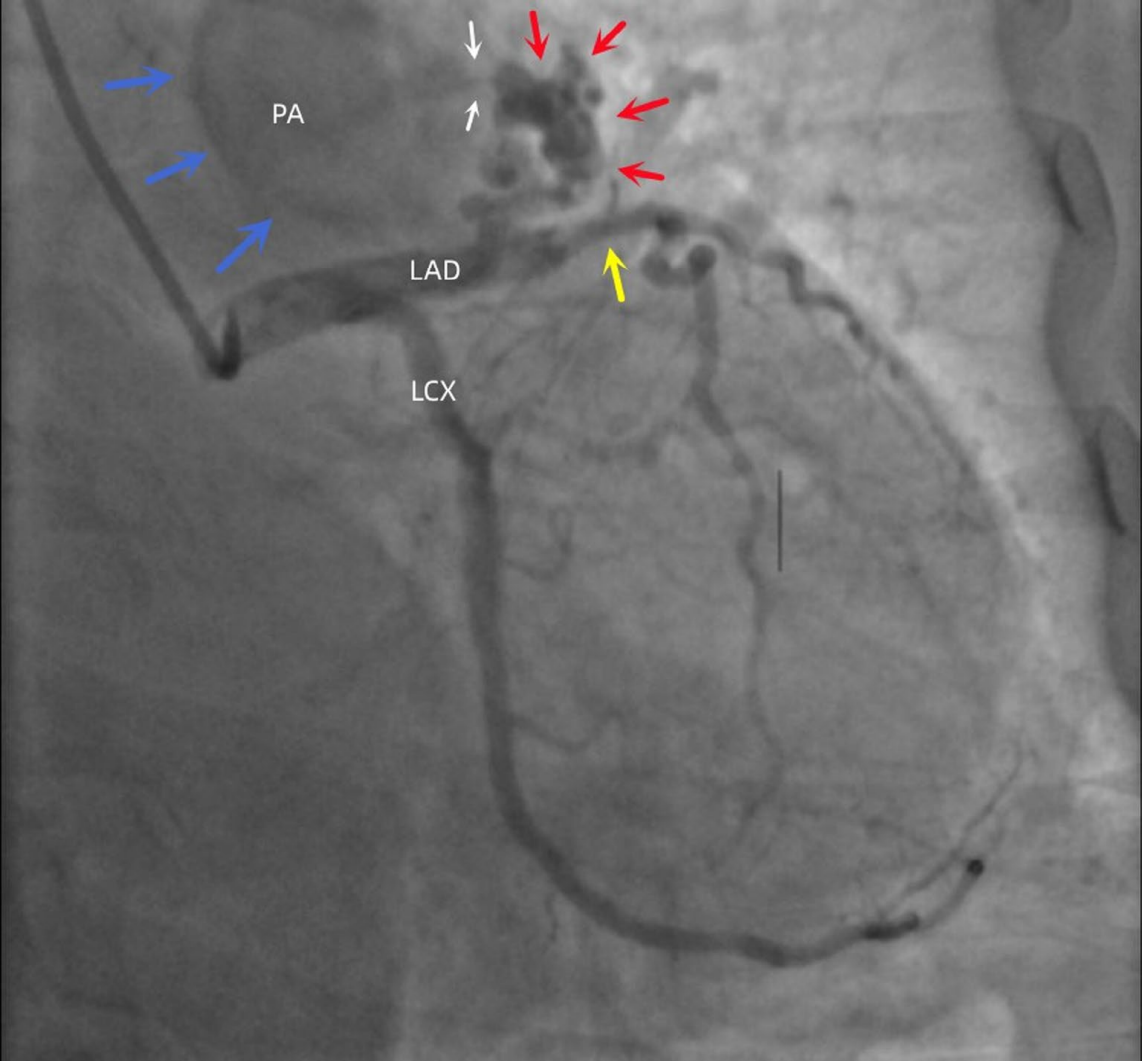
CAG revealed a 50% stenosis in the middle segment of the LAD (yellow arrow), with a cluster of malformed vessels originating from the proximal segment (red arrow), and fine blood flow (white arrow) is observed jettisoning towards the PA (blue arrow). CAG, coronary angiography; LAD, left anterior descending; LCX, left circumflex artery; PA, pulmonary artery.

However, the CAG demonstrated about a 50% luminal narrowing in the proximal to mid‐segments of the left anterior descending (LAD), and there was a coronary arteriovenous fistula originating from the proximal part of the LAD artery, which drained into the pulmonary artery (PA) (Figure 1). So we confirmed the diagnoses of coronary heart disease (CHD) and CPAF.

Considering the degree of coronary stenosis did not meet the criteria for interventional treatment and the shunt flow from the anomalous vessel was minimal and not sufficient to cause symptoms, we decided to manage her with medical therapy and not to intervene on the CPAF.

However, the patient continued to experience symptoms of chest discomfort and dizziness postoperatively. Although the effects of medication take time to manifest, to rule out other conditions, we proceeded to conduct further examinations.

A 24‐h Holter monitor showed no major issues. To clarify the extent of myocardial ischemia, we proceeded with additional diagnostic evaluations by cardiac magnetic resonance imaging (MRI) and adenosine stress emission computed tomography (ECT). The results showed hypertrophic changes in the ventricular walls due to hypertension, without significant ischemic signs.

**FIGURE 2:**
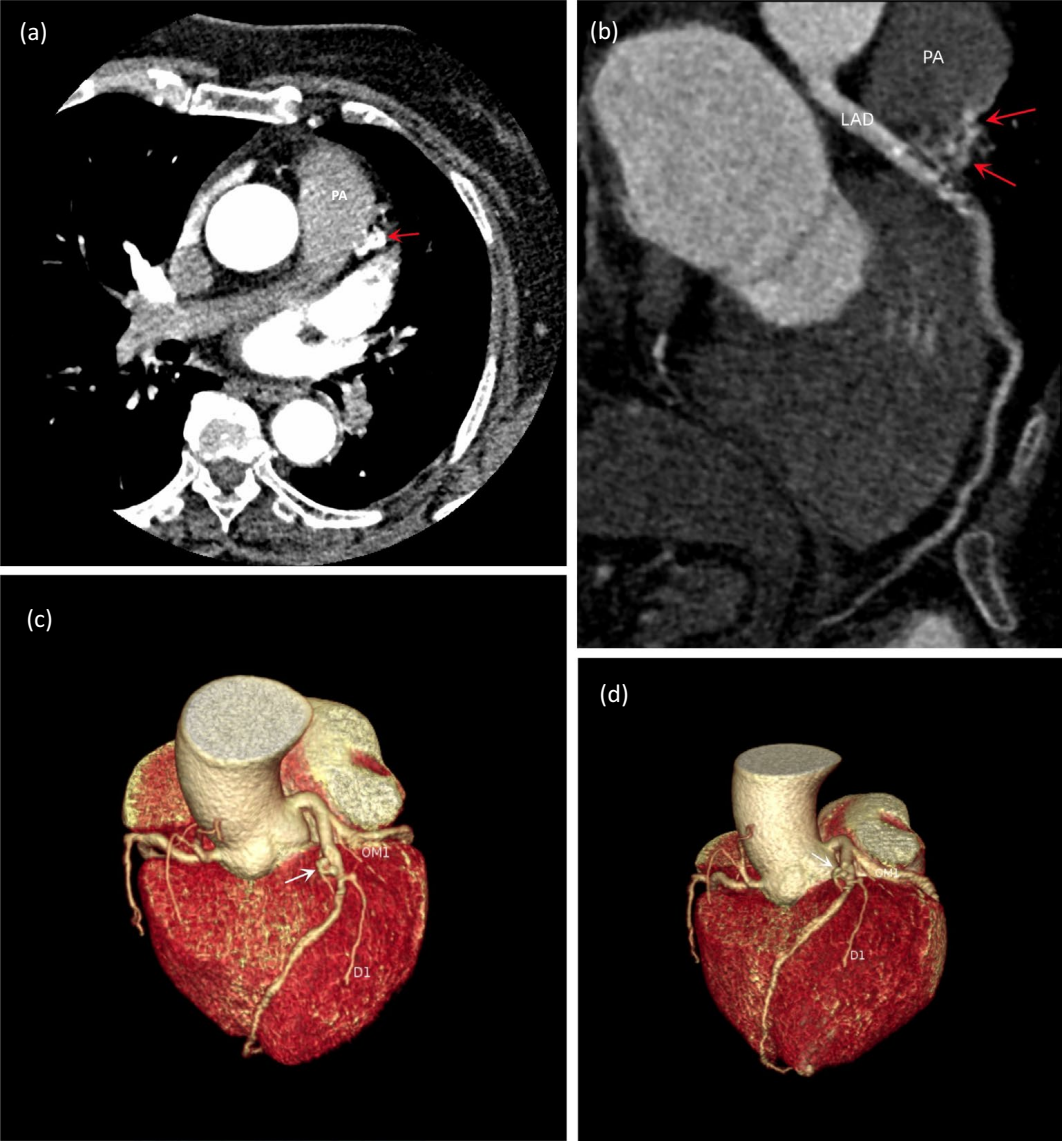
(a) The CT shows an abnormal vascular mass (red arrow) with a hint of high‐density at the PA connection, indicating minor blood flow ejection into PA. (b) Curved reconstruction CT also reveals near the PA, minimal high‐density suggests slight blood flow via the fistula (red arrow). (c, d) The CTA revealed 3D images of the fistula (white arrow), but there was no visualization of the PA. CTA, Computed Tomography Angiography; D1, first diagonal branch; LAD, left anterior descending; OM1, first obtuse marginal branch; PA, pulmonary artery.

Postprocessing 3‐D image of coronary computed tomography angiography (CTA) confirmed the CAG findings, indicating mild to moderate stenosis of the LAD, and also revealed the presence of the anomalous CPAF. Interestingly, the PA is not visualized in the vascular reconstruction images, which we suspect is due to the minimal blood flow from the fistula draining into it, and the contrast medium cannot fully fill (Figure 2a–d).

### 3.3 Treatment

Given these findings, we concluded that the small fistula did not cause significant symptoms or severe shunting. Following the consultation advice from the neurologist, we conducted a brain MRI, which revealed small vessel arteriosclerotic leukoencephalopathy; her symptoms are partly attributed to a neurological condition.

We advise her to continue the pharmacological treatment for CHD and hypertension. The specific medication regimen includes: Aspirin 100 mg daily, Atorvastatin 20 mg daily, Nifedipine 30 mg daily, Sacubitril‐valsartan 200 mg daily, and Metoprolol 23.75 mg daily. Additionally, continue oral administration of repaglinide 1 mg daily for glycemic control. All aforementioned medications are prescribed for chronic use, with explicit instructions that dosage adjustments must be conducted under medical supervision.

Additionally, comprehensive health education was provided to the patient, emphasizing adherence to a low‐sodium, low‐fat diabetic diet for metabolic optimization, maintenance of structured daily routines, engagement in moderate physical activity, and avoidance of physical exhaustion or emotional stressors. Scheduled monitoring protocols include regular laboratory assessments (complete blood count, lipid profile, hepatic/renal function, coagulation studies, etc.) and cardiovascular surveillance (ECG, TTE, carotid ultrasonography, etc.), with mandatory close outpatient follow‐up at our institution's cardiology clinic for ongoing therapeutic evaluation and adjustments. Recurrent CAG re‐evaluation as clinically indicated.

## 4 Outcome and Follow‐Up

During the telephone follow‐up 3 months later, the patient reported a significant improvement in symptoms, with chest discomfort only occurring during more intense physical activities, but she still occasionally experienced dizziness. We advised her to continue taking the medications and to schedule an appointment with a neurologist to address the dizziness. We will continue to observe her follow‐up results at subsequent intervals (6 months, 1 year, and beyond post‐discharge) and maintain an optimistic outlook.

## 5 Discussion

Coronary artery fistulas (CAF) are abnormal connections between a coronary artery and another cardiovascular structure, such as a cardiac chamber, the superior vena cava, or the coronary sinus [1]. Congenital CAF are rare conditions in cardiovascular diseases, which account for approximately 0.3% of all congenital heart diseases [2]. Our patient was incidentally found to have a CPAF during CAG, with the fistula connecting the left anterior descending artery and the main PA. This type of CAF is estimated to have a prevalence of about 0.17% [3]. Most patients with CAFs are asymptomatic, but a minority with significant blood shunting may have symptoms like mild dyspnea, chest discomfort, dizziness, and angina [4]. Severe cases can lead to heart failure, pulmonary congestion, and myocardial infarction [5]. This is also why we did not initially attribute the patient's symptoms to CPAF.

Various imaging modalities can provide diagnostic evidence, with CAG being the first‐line diagnostic method for coronary artery fistulas [6]. ECT and cardiac MRI also have additional diagnostic value [6, 7]. In addition, coronary CTA can vividly display the 3D structure of blood vessels [8]. But if the blood flow is too minimal, it could lead to the vessel not being visualized, resulting in a potential missed diagnosis, as in this patient's case. Although a 3D image of the anomalous vessel was presented, the blood flow into the PA was so minimal that the PA did not opacify.

CPAF treatment options include medical management and surgery [9]. The main reasons for our choice of medication treatment are as follows: (1) We considered that the patient's chest discomfort and palpitations were mainly caused by CHD. Based on the existing examination results (CAG, CTA, etc.), it seems that this CPAF is not likely to cause the current symptoms, or at most, it is just one of the factors that exacerbate the existing symptoms. Compared to occluding a CPAF with a small shunt flow, the LAD (with a 50% stenosis) deserves treatment more. (2) Patients with obvious clinical symptoms or imaging suggesting significant shunting and excessive volume load are recommended for treatment [2]. Currently, there is no reliable evidence that treating this type of fistula would benefit the patient's prognosis. The academic community has not reached a consensus on whether to treat such lesions [10, 11]. (3) We discussed with the patient the different treatment approaches between medical therapy and surgical intervention, and the patient expressed a preference for initial conservative management, with surgery reserved should pharmacotherapy prove ineffective.

Reflecting on this case, we pondered whether opting for CAG prior to comprehensive non‐invasive imaging was too aggressive. From the results, coronary CTA has been sufficient to help us confirm the diagnosis of CHD and CPAF, but CTA failed to clarify which vessel the fistula ultimately drains into, and it seems that further evaluation with CAG is still needed. Additionally, the presence of a CPAF often leads to hemodynamic dysfunction, which could potentially accelerate the progression of LAD stenosis. Based on the aforementioned clinical considerations, a rigorous reassessment of the risk–benefit profile may warrant evaluation of whether early surgical intervention could confer superior clinical outcomes relative to conservative management. However, from the follow‐up observations, the effectiveness of our conservative treatment is commendable. Furthermore, some relatively rare diseases should have been considered at the time, such as vascular abnormalities of the spine, cardiac syndrome X, and others [12, 13].

## Author Contributions

**Jia‐jun Zhang:** conceptualization, data curation, investigation, methodology, software, validation, writing – original draft. **Xiao Li:** data curation, formal analysis, methodology, software, writing – review and editing. **Jin‐long Zhang:** data curation, formal analysis, resources, software, validation, visualization. **Hai‐tao Zhang:** project administration, resources, supervision, validation. **Fei‐fei Su:** conceptualization, funding acquisition, methodology, project administration, resources, supervision, validation, writing – review and editing.

## Ethics Statement

The study was conducted in accordance with the principles of the Declaration of Helsinki. The patient provided written informed consent to participate in this study and agreed to the publication of all data and images. The approval was provided by the Medical Research Ethics Committee of Chinese People's Liberation Army Air Force Medical Center.

## Conflicts of Interest

The authors declare no conflicts of interest.

## Data Availability

The data that support the findings of this study are available from the corresponding author upon reasonable request.
